# Perineal body squamous cell carcinoma treated with radical radiotherapy – a case report

**DOI:** 10.3332/ecancer.2023.1534

**Published:** 2023-04-20

**Authors:** Nikunj Patil, Shirley Lewis, Chethana B Udupa, Rajagopal K V, Krishna Sharan

**Affiliations:** 1 Department of Radiotherapy and Oncology, Kasturba Medical College, Manipal Academy of Higher Education, Manipal 576104, Karnataka, India; 2 Department of Pathology, Kasturba Medical College, Manipal Academy of Higher Education, Manipal 576104, Karnataka, India; 3 Department of Radiology, Kasturba Medical College, Manipal Academy of Higher Education, Manipal 576104, Karnataka, India

**Keywords:** perineal body, perianal cancer, radiotherapy, IMRT, case report

## Abstract

**Introduction:**

Perianal tumours are a rare site of malignancy, and tumours primarily involving the perineal body without vaginal and anal canal involvement are uncommon.

**Case summary:**

A 67-year-old woman presented with a lesion involving the perineum and rectovaginal septum without extension into vaginal or anorectal mucosa and with skip lesions in the vulva. Biopsy was confirmative of squamous cell carcinoma, with p16 positive. A complete metastatic workup with MRI of the pelvis and CECT thorax and abdomen was done. She was diagnosed with perianal carcinoma stage cT2N0M0 Stage II (American Joint Committee on Cancer 8th Edition of Cancer Staging) since the lesion reached the anal verge. Given the location of the tumour (perineal body), comorbidities and advanced age, she received radical radiotherapy with an intensity-modulated radiotherapy technique – 56 Gy in 28 fractions with the intention of organ preservation. The response assessment with MRI at 3 months showed a complete tumour response. She has been disease-free for 3 years and is on regular follow-ups.

**Conclusion:**

Isolated perineal body squamous cell carcinomas are unusual, and synchronous vulvar skip lesion makes this case unique. Radical radiotherapy achieved organ preservation with tumour control and minimal toxicity in an elderly frail patient.

## Introduction

Tumours of the anal canal, perineum and perianal skin are infrequent. As per GLOBOCAN 2020, the incidence of anal malignancies is 0.3%, with a slight female preponderance [1]. Carcinoma of the perianal skin or anal margin accounts for 12%–23% of anal canal cancers [2, 3]. Anal canal cancers have a high risk of lymphatic spread. Tumours in the perianal region are akin to skin cancer. Squamous cell carcinoma is the most common histology type, followed by adenocarcinomas. Other histologies are verrucous carcinoma (giant condyloma), condyloma acuminatum, Bowen’s disease, Paget’s disease, melanoma, basal cell carcinoma and metastases [4].

Tumours arising entirely from the perineal body are unique. Their stage grouping can be either vulvar or perianal. The American Joint Committee on Cancer (AJCC) defines tumours that arise within the skin at or distal to the anal verge, within 5 cm of perianal cancers or anal margin. In contrast, tumours in the perineum that do not reach the anal verge are considered vulvar [5]. However, in the absence of the vulva, vagina or anal canal, involvement with lesions predominantly within the perineal body and rectovaginal septum would pose a diagnostic dilemma for staging and site assignment. This type of presentation is not very typical of perianal carcinoma as it is superficial and shows proliferative growth. Surgery is the most common mode of treatment for early perianal cancers. Radiotherapy is considered in cases with margin positivity and recurrent or locally advanced lesions.

We present a case of an elderly woman with a lesion involving the perineum with skip lesions in the vulva treated with radical radiotherapy alone. The peculiarity of this case lies in the tumours’ location as it was in the perineal body without the involvement of mucosa of the anal canal or vagina, along with a skip lesion over the labia majora. Radiotherapy enabled an organ preservation approach for this patient with a complete response, and local control was maintained for over 3 years.

## Case summary

**Figure 1.**
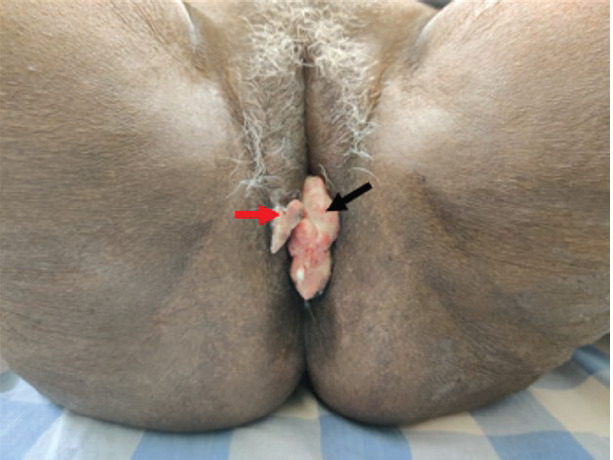
Clinical picture of patient at presentation showing the perianal lesion (black arrow) with separate skip lesion in vulva (red arrow).

A 67-year-old post-menopausal woman presented with a chief complaint of progressive growth in the perianal region for 5 months. Her comorbidities include asthma and hypertension. On local examination, there was a 4 × 3 × 1.5 cm ulcero-proliferative growth overlying the perineum, extending from the fourchette to just short of the anal verge posteriorly. The lesion was pink in colour, firm in consistency and fixed to the perineum. Another lesion of 1 × 1 cm was noted over the right labia (Figure 1). A complete per speculum and vaginal examination, along with a digital rectal examination, revealed no extension into the vaginal or anal/rectal mucosa; however, the rectovaginal septum was thickened with no clinically palpable inguinal lymphadenopathy. The systemic examination results were normal and revealed no signs of metastases.

**Figure 2.**
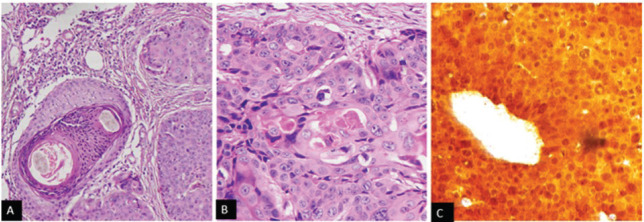
(a): Photomicrograph showing atypical squamous cell in nests, cords and sheets. (b): Large cells with eosinophilic cytoplasm, elongated nucleoli with keratins pearls and have diffuse cytoplasmic and (c): nuclear positivity for p16.

**Figure 3.**
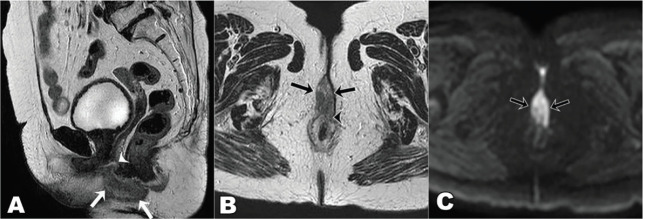
T2 weighted MRI images sagittal view (a): axial view (b): diffusion-weighted axial image (c): at presentation showing T2 hyperintense mass lesion (arrows in a and b) showing diffusion restriction (arrows c) in the ano-vaginal space of the urogenital triangle with loss of fat planes with the vagina (arrowhead in a) and anterior external anal sphincter (arrowhead in b).

**Figure 4.**
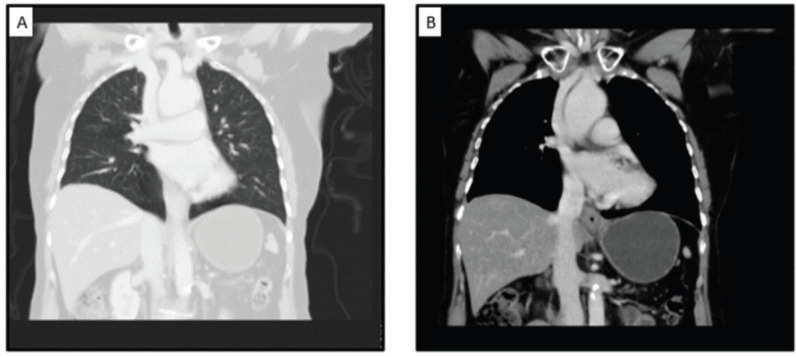
CECT showing (a): non-metastatic status of the patient in the thorax and (b): no lymphadenopathy.

She underwent diagnostic evaluation with biopsy, complete blood counts, liver function, kidney function test, viral markers (Human Immunodeficiency virus, Hepatitis B antigen and Hepatitis C), magnetic resonance imaging (MRI) of the pelvis and contrast-enhanced computed tomography (CECT) of the chest and abdomen. All blood test results were within normal limits, and the viral markers were negative. The biopsy was confirmative of squamous cell carcinoma with p16 positive on immunohistochemistry (Figure 2). The MRI of the pelvis revealed a T2 hyperintense lesion in the ano-vaginal space of the urogenital triangle extending anteriorly up to the posterior wall of the vagina, and posteriorly up to the external anal sphincter at the anal verge, without any apparent mucosal infiltration (Figure 3). There was a loss of fat planes in the external anal sphincter. Fat planes were maintained with the urethra, and puborectalis, ischiorectal fossa and pelvic sidewalls were uninvolved. There was no pelvic or inguinal lymph node enlargement. CECT thorax, abdomen and pelvis showed no distant metastases (Figure 4). Her final diagnosis was established as perianal carcinoma stage cT2N0M0 Stage II per the AJCC 8th Edition Tumor, node metastasis staging system since the lesion reached the anal verge. The case was discussed by the multidisciplinary tumour board. Surgery would entail abdominoperineal resection (APR)/posterior exenteration with a permanent colostomy to achieve negative margins. The patient was offered both the options of surgery and radiotherapy; however, given the tumour’s location (perineal body), comorbidities (asthma and hypertension), frailty and advanced age of the patient, we offered her radical radiotherapy to achieve organ preservation and surgery as a salvage option. Concurrent chemotherapy was not administered due to the patient’s frailty and preferences to avoid chemotherapy.

**Figure 5.**
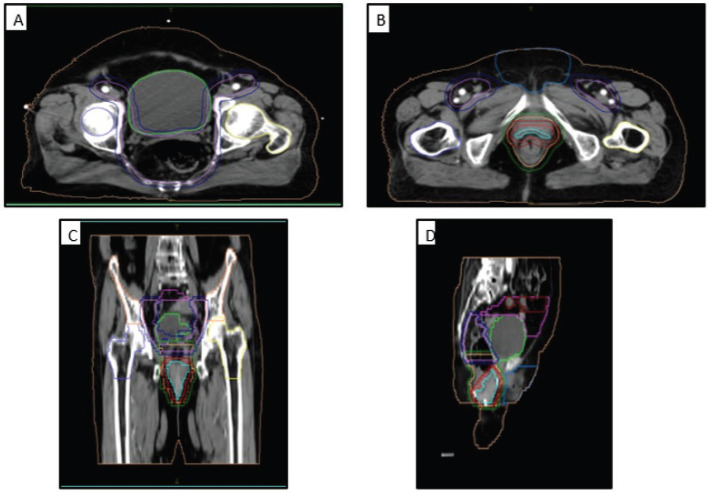
Contouring volumes of the pelvic lymph nodes in (a): the axial view, (b): the gross disease, (c): coronal view and (d): sagittal view. GTV: cyan, CTV-56: bright red, CTV-50.4: peach, CTV-42: lavender, PTV-56: red, PTV-50.4: green, PTV-42: dark blue, Lt femur: yellow, Rt femur: blue, Ext genitalia: light blue, bladder: lime green, bowel bag: pink, small bowel: red, iliac crest: orange, external: beige.

**Figure 6.**
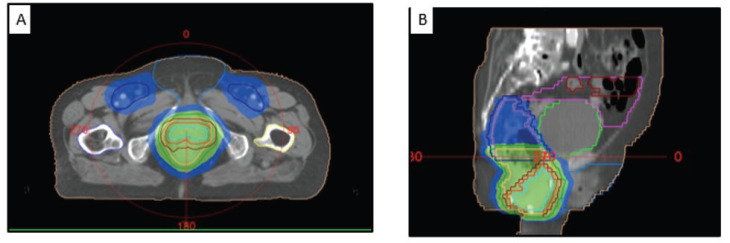
(A) Axial and (B) sagittal CT images showing the dose coverage of the gross disease and nodal volumes.

**Figure 7.**
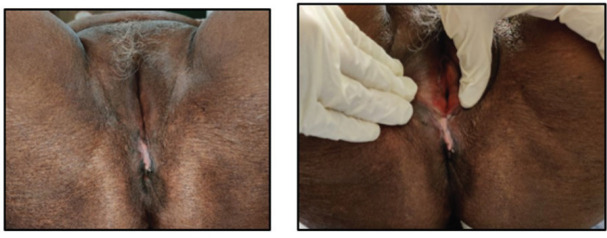
Clinical picture of the patient at the conclusion of treatment showing regression in the size of the lesion.

As the tumour was reaching the anal verge, we treated the patient with the anal canal squamous cell cancer protocol. The patient was treated with intensity-modulated radiotherapy (IMRT) with curative intent. She underwent a radiotherapy planning computed tomography (CT) scan in a supine position with pelvic thermoplastic mould with full bladder protocol. The high-risk clinical target volume (CTV) included lesions with a margin of 1 cm, and the low-risk CTV included the pelvic and bilateral inguinal nodes. The planned dose was 56 Gy in 28 fractions for high-risk CTV and 42 Gy in 28 fractions (EQD2 40 Gy) for low-risk CTV. The dose constraints for all organs were met as per radiation therapy oncology group (RTOG) 0529 except external genitalia, as the lesion in the right labia was included in high-risk CTV (Table 1) [6]. She was treated with a volumetric modulated arc therapy technique using 6 MV photons over 5.5 weeks, 5 days per week (Figure 5 and 6). She tolerated the treatment well, with grade 2 dermatitis and enteritis, without any undue interruption. At completion, a single 1.5 × 1 cm lesion was present at the posterior fourchette of the vagina, with a complete response of the lesions over the labia (Figure 7).

**Figure 8.**
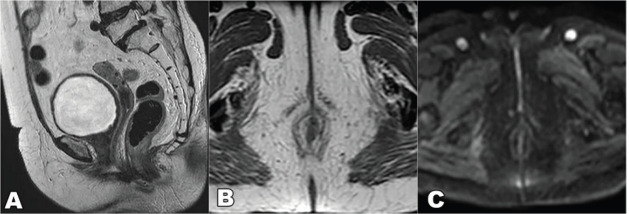
T2 weighted MRI sequence, (a): sagittal, (b): axial and (c): DWI sequence showing complete resolution of the lesion.

She was followed up monthly for the first 3 months and every quarterly thereafter. There was progressive regression in the size of the lesion. At 3 months after treatment, there was a complete resolution of the lesion, with post-treatment hypopigmentation. Re-assessment MRI revealed complete resolution of the tumour (Figure 8). The patient has completed 3 years of surveillance to date with no toxicity, except hypopigmentation, and remains clinically disease-free. She will be monitored on regular follow-up for 5 years to assess for local recurrence or new primary in the vulva/vagina/anal canal. Written informed consent was obtained from the patient.

## Discussion

Malignancy arising from the anal margin is uncommon in anal canal tumours. By definition, perianal cancers do not involve the anal canal mucosa and occur within a diameter of 5 cm from the anal verge. Since they originate from the skin and are often superficial, perianal cancers behave like skin cancers. They were staged as skin cancers until the 8th edition of AJCC, which includes them in anal cancer staging. Perianal skin can develop many benign lesions like haemorrhoids, fissures, cysts or fistula [7]. Due to the rarity and non-specific presentation, these tumours are often neglected, misdiagnosed, and prone to delayed diagnosis. When the lesions are less than 2 cm, it is easy to ascertain the origin and classify them as vulva, perianal or anal. However, in larger lesions invading other structures like the anal canal, identifying the origin becomes challenging. Such lesions are often classified and treated as anal canal cancers. The uniqueness of this case was in the fact that the entire lesion was in the perineal body without involving anal mucosa with skip lesions in the vulva compared to a pure perianal which is superficial (surgical excision amenable) or anal with perianal extension. The location is challenging to assign as either anal perianal or vulvar cancer, as there is no terminology for perineal body cancer. The presence of skip metastases is not incorporated into the staging of either the perianal/anal or vulvar. Despite the skip lesion in the vulva, due to the lesion reaching the anal verge, we staged it as perianal cancer.

Human papillomavirus (HPV) infection is the leading factor for developing anal canal cancers and anal preneoplastic lesions [8, 9]. Over 90% of cervical and anal canal cancers and about 40%–50% of other genital cancers (penis, vulva and vagina) are attributed to HPV [10]. Skip lesions from anal canal cancers involving the lower and mid rectum are known to occur [11]. Vulvar recurrences or presentations in anal canal cancer are rare, and the radiotherapy protocols with IMRT propose genitalia sparing during planning. Koeck *et al* [12] describe three cases with genitalia involvement at presentation and one case of vulvar recurrence post-treatment of anal cancer. The authors propose that perigenital direct infiltration or lymphatic spread is the principal reason for genitalia involvement. However, the lesions were locally advanced with inguinal node involvement, which supports the hypothesis. Bagshaw *et al* [13] report two vulval recurrences in a cohort of 52 patients of anal cancer treated with chemoradiation. The authors suggest that in the presence of bulky primary and inguinal/pelvic nodes, the in-transit dermal lymphatics are subclinically involved giving rise to vulval recurrence. In the present case, the skip lesion in the vulva was superficial with T2 primary and in the absence of nodal involvement. We hypothesise that the vulvar skip lesion may indicate an anogenital field cancerisation effect from HPV exposure. Gilbert *et al* [14] have shown that initial HPV-related cancer or premalignant lesion is at risk for subsequent development of second HPV-related cancer. We could not find any reports of skip lesions from perianal cancer presenting in the vulva in the literature. While the incidence of synchronous HPV tumours of genitalia with the anal canal is unknown, there are case reports of synchronous HPV tumours of the cervix and anal canal [15, 16].

Treatment aims to cure the patient while preserving anal function, thus avoiding a permanent colostomy and affording a better quality of life. Traditionally, treatment consists of either local excision with or without adjuvant radiotherapy or APR in advanced cases [17]. For early T1-2N0 lesions, local excision is the preferred choice for complete excision with negative margins. The perianal anatomy limits a large margin or repeated excision due to the risk of morbidity. Hence, a majority require adjuvant radiotherapy for positive or close margins [18, 19]. The Personalising anal cancer radiotherapy dose trial (personalising radiotherapy dose in anal cancer, ISRCTN88455282) Anal cancer trial 3 trial is an effort to standardise local excision and adjuvant radiotherapy practice for T1N0M0 anal cancer [20]. The trial aims to standardise adjuvant treatment for patients who undergo surgery and have postoperative margins <1 mm. The trial is currently ongoing. It is extremely difficult to achieve a clear margin in patients with an extensive perianal disease; hence, these patients undergo adjuvant chemoradiation. For large lesions in perianal skin invading the anal sphincter, surrounding organs and bulky nodes, the preferred treatment is radical chemoradiation, similar to that for tumours arising from the anal canal. Randomised trials demonstrate the superiority of chemoradiotherapy over radiotherapy alone in terms of disease-free survival, local relapse, and colostomy-free survival [2, 21]. Early-stage anal cancers account for only 10%–15% of patients and are under-represented in trials. The retrospective series report varied outcomes for patients with T1-2N0M0 disease with Radiotherapy alone, with some favouring Radiotherapy alone and others preferring chemoradiation [22, 23]. Due to the patient’s advanced age, frailty and comorbidities, the patient was planned for radical radiotherapy alone. Several retrospective and prospective cohort studies support the safety of IMRT in conjunction with concurrent chemotherapy for anal Squamous cell carcinoma, with promising early efficacy results [6, 24–26]. Its benefits in reducing acute toxicities (small and large bowel, genitalia, pelvic skin and soft tissues) are apparent.

We could not find any case reports in the literature on perineal body carcinoma treated with radiotherapy. This case highlights the favourable response of radiotherapy alone for perineal body carcinoma with vulvar skip lesions in frail elderly patients. Few case reports reported on perineal cancer (recurrent perineal and Paget’s disease) treated with radical radiotherapy alone [27, 28]. While chemoradiation is the standard of care, elderly frail patients are often unable to tolerate full-dose chemoradiation. Low-dose chemoradiation has been attempted in this population with 30 Gy in 15 fractions resulting in local control of 73% [29]. The results from large institutional series have shown a local failure to be low in T2 lesions (high grade and ≥4 cm) treated with high-dose chemoradiation of 63 Gy [25]. Patients treated with a dose of 55–62 Gy with radiotherapy alone in other retrospective reports have resulted in local control of 74%–90% for early lesions [30, 31]. Hence, in the present case, we opted to treat the patient with a high dose to the primary of 56 Gy instead of 50.4 Gy (as in RTOG 0529) for the T2N0 lesion. Early anal or perianal lesions (T1–T2 N0) in frail elderly or unfit patients may be treated with high-dose radiotherapy alone with acceptable local control.

## Conclusion

Isolated perineal body squamous cell carcinomas are unusual, and synchronous vulvar skip lesion makes this case unique. Radical radiotherapy achieved organ preservation with tumour control with minimal toxicity in an elderly frail patient.

### Patient perspective

I noticed a lump near the anus for 2–3 months. I saw my family doctor, who told me that this looks suspicious, and he will not be able to treat it. He referred me to a larger hospital close to my hometown. I hoped they could treat it; otherwise, it would remain as is. I was explained about the mass near the anus and was told to get blood tests with scans. After looking at the scans, the doctor told me about the disease status and available treatment options. I was worried when they told me that I was suffering from cancer. I was not scared. I was ready to face anything that comes my way. I was anxious as I did not know what the future held. When I visited the radiation oncologist, I was explained about radiotherapy, the course of treatment and that my disease could be treated. This brought ease to my mind, as I was not undergoing surgery and hoped that the disease will disappear with radiation. Although my treatment would last for 5.5 weeks, I felt if it had to be done to get better, I will do it.

The treatment started within the next couple of days. I followed up with the doctor every week during the treatment. Towards the end, I noticed changes in the skin in the anal region, which slowly went away after treatment. I was pleased to see the tumour shrink during treatment, which brought me extreme joy and hope. They asked me to do the scans after 3 months, and the tumour had completely disappeared. I was delighted with this news. I was hopeful for the future. Since then, I have not felt any mass in that region. I have been back to my normal routine at home and I am grateful for all the care I have had. I am following up with the doctor. I am well and have not experienced any pain or discomfort after treatment.

## List of abbreviations

AJCC, American Joint Committee on Cancer; APR, abdominoperineal resection; CECT, contrast-enhanced computed tomography; CTV, clinical target volume; HPV, human papillomavirus; IMRT, intensity-modulated radiotherapy; MRI, magnetic resonance imaging; RTOG, radiation therapy oncology group.

## Conflicts of interest

None.

## Funding

No funding was received.

## Figures and Tables

**Table 1.** Showing the doses received by planning target volumes and organs at risk.

Organ	Planning constraints	Dose received
PTV 56 Gy	V 95% ≥ 95%	96%
PTV 42 Gy	V 95% ≥ 95%	95%
Bowel (small and large)	V 45 < 20 cc V 35 < 150 cc V 30 < 200 cc	2.9 cc 79 cc 141 cc
Femoral heads	V 44% < 5% V 40% < 35% V 30% < 50%	0% 15% 32%
External genitalia	V 40% < 5% V 30% < 35% V 20% < 50%	20% 41% 81%
Bladder	V 50% < 5% V 40% < 35% V 35% < 50%	0% 10% 29%
